# The Different Facades of Retinal and Choroidal Endothelial Cells in Response to Hypoxia

**DOI:** 10.3390/ijms19123846

**Published:** 2018-12-03

**Authors:** Effat Alizadeh, Parviz Mammadzada, Helder André

**Affiliations:** 1 Department of Clinical Neuroscience, St. Erik Eye Hospital, Karolinska Institutet, 11282 Stockholm, Sweden; alizadehe@tbzmed.ac.ir (E.A.); Parviz.Mammadzada@ki.se (P.M.); 2 Department of Medical Biotechnology, Faculty of Advanced Medical Sciences, Tabriz University of Medical Sciences, 5166/15731 Tabriz, Iran

**Keywords:** choroidal endothelial cells, retinal endothelial cells, hypoxia, angiogenesis, differential expression

## Abstract

Ocular angiogenic diseases, such as proliferative diabetic retinopathy and neovascular age-related macular degeneration, are associated with severe loss of vision. These pathologies originate from different vascular beds, retinal and choroidal microvasculatures, respectively. The activation of endothelial cells (EC) plays pivotal roles in angiogenesis, often triggered by oxygen deficiency. Hypoxia-inducible factors in ECs mediate the transcription of multiple angiogenic genes, including the canonical vascular endothelial growth factors. ECs show notable heterogeneity in function, structure, and disease, therefore the understanding of retinal/choroidal ECs (REC; CEC) biochemical and molecular responses to hypoxia may offer key insights into tissue-specific vascular targeting treatments. The aim of this review is to discuss the differences spanning between REC and CEC, with focus on their response to hypoxia, which could provide innovative and sustainable strategies for site specific targeting of ocular neovascularization.

## 1. Introduction

Ocular neovascularization is often associated with aberrant formation of immature blood vessels, which can lead to sight-threatening conditions when located in the light-sensing tissue of the eye, the retina. Currently, two retina-associated disorders with high incidence in the Western world include proliferative diabetic retinopathy (PDR) and age-related macular degeneration (AMD). Both pathologies display clinical manifestations of distorted vision and, in severe progressive cases, even vision loss [1,2]. In diabetic patients, new blood vessels sprout from the retinal vasculature leading to PDR [3,4]. Wet or neovascular AMD (nAMD) represents the clinical pathology when angiogenesis triggers the choroid capillaries to invade the subretinal compartment, through the Bruch’s Membrane (BM), a process termed choroidal neovascularization (CNV) [5]. Although, the molecular and cellular mechanisms orchestrating the neoangiogenesis in PDR and nAMD diseases have not been fully understood, the presence of a strong stimulatory factor, hypoxia, has been suggested in both [4].

Hypoxia promotes endothelial cells (ECs), the inner lining cells of blood vessels’ lumen, to enter angiogenesis. Hence, the microvascular ECs generated from the retina (REC) or the choroid (CEC) may display different phenotypes that illustrate the specific requirements of the corresponding tissues and their pathologic involvement [6]. Besides, the heterogeneity of ECs from different species, tissues, and even from the same tissue have been described which could denote their differential contribution in different diseases [7]. In broad terms, neoangiogenesis represents the formation of new blood vessels from the existing vasculature, and is mediated by EC proliferation, re-arrangement of extra-cellular matrix (ECM), EC migration, and tubulogenesis [8]. The newly formed blood vessels are natively fragile, resulting in fluid leakage within the retinal tissues, which can lead to permanent damage to the photoreceptors and ultimately vision loss in untreated cases [9]. Molecular determinations have confirmed that oxygen deprivation in the eye due to different pathologic conditions could be pivotal in the progress of retinal vascular pathologies [4,10,11]. In fact, hypoxia triggers a series of responses that direct the adaptation of ECs to the stress situation. Adaptation to hypoxia starts via a switch to anaerobic metabolism (glycolysis and lactate), transcriptional upregulation of several genes including angiogenesis growth factors such as vascular endothelial growth factor (VEGF), upregulation of red blood cells proliferation stimulator (erythropoietin; EPO), and several other related functions [12]. These transcriptional tunings are facilitated by a family of transcription factors, the hypoxia-inducible factors (HIFs) [13]. HIFs are composed of two different proteins including α- and β-subunits, which form a complex upon oxygen deprivation and recognize hypoxia-response elements (HREs) on regulatory areas of corresponding hypoxia-mediated genes [11]. The main downstream function of hypoxia signaling is promotion of angiogenesis in retinal and choroidal microvasculatures, with the aim of compensating the low oxygen tension. Existing treatments have adequately addressed angiogenesis, yet some patients fail to respond to treatment and considerable side effects can emerge from frequent administrations. Description of the molecular networks involved in hypoxia-mediated neovascularization can lead to the recognition of new targets for therapeutic interventions.

The angiogenesis of diverse tissues may not be mediated by the same stimulatory effectors and downstream signaling molecules, which suggests that the response of ECs from different tissue sources to hypoxia conditions could be unique to their origin [4]. A few studies have focused on the comparative transcriptome and proteome expression of human or animal derived REC and CEC exposed to VEGF or inflammatory stimuli [3,4,14,15,16]. Yet, to the best of our knowledge, only one report exists to date in the literature regarding hypoxia exposure of human-derived REC and CEC and exploring their responses [6]. Research focused on comparative gene and protein phenotypes of REC and CEC observed that most proteins were expressed in the same pattern between REC and CEC, in accordance with their similar microvascular phenotype. On the other hand, it has been suggested that angiogenesis inside the retina might be organized by different contrivances to those controlling choroidal neovascularization [17,18]. As a result, choroidal and retinal ECs may have dissimilar expression profiles upon stimulation with oxygen deprivation, thus the understanding of their regulation mechanism renders the development of successful discriminant therapies against PDR and nAMD applicable in the clinic [6]. In this manner, isolation of REC and CEC, as well as comparative studies of gene and protein expression of their responses to hypoxia, could be beneficial in terms of providing new insights for targeted gene therapy without affecting other vascular beds. The present review is aimed at outlining the reported differences in molecular phenotypes between ECs isolated from retinal or choroidal origin at baseline and in response to hypoxia microenvironment exposure, as well as other external stimuli.

## 2. The Microvascular Architecture in the Posterior Eye

**Figure 1:**
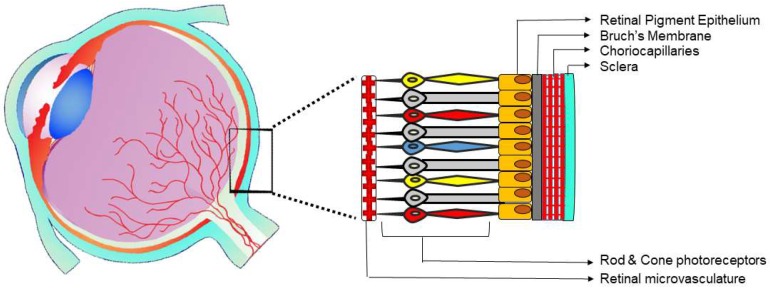
Schematic representation of the two microvascular beds in the posterior eye segment. The light-sensing tissue of the eye, retina, is irrigated by the retinal microvasculature. The choriocapillaries are located in the choroid, between the sclera and Bruch’s membrane, and supply the outer retina. As example, choriocapillaries are the vessels affected in neovascular age-related macular degeneration (nAMD), while the retinal microvasculature is responsible for the pathologic angiogenesis of proliferative diabetic retinopathy (PDR).

The posterior segment of the eye comprehends the retina, choroid, and optic nerve, yet also the vitreal compartment filled with vitreous humor [19]. Innately, two different vasculature beds feed the eye posterior tissues including choriocapillaries and retinal micro-vessels (Figure 1).

### 2.1. Developmental Vascularization of the Posterior Eye Segments

During embryonic development, blood vessels form through recruitment of progenitor vascular cells which undergo differentiations and remodeling to form the primary vasculature [20]. Following vasculogenesis, vascular networks expand through angiogenesis, or the formation of new blood vessels from pre-existing ones, which comprise two distinct types: sprouting angiogenesis where ECs branch out from the existing vessels and recruit pericytes for vessel formation; and intussusceptive angiogenesis, a process through perforation of vessels by specific pillars generated by EC and pericytes leading to splitting of the vessel. The choriocapillaries are thought to employ intussusception as a mechanism of vascular growth and remodeling during development as opposed to the development of retinal vasculature which occurs mainly through vascular sprouting [21,22]. There are distinct differences in the development of the two vascular beds. On the fourth week of gestation in humans, the undifferentiated mesoderm surrounding the optic cup begins to differentiate and form ECs adjacent to the retinal pigment epithelium (RPE). These early vessels are the precursors of the choriocapillaries. The choroidal capillary network becomes almost completely organized by the eighth gestational week. In contrast, the formation of retinal vasculature begins at a later stage in gestation. Until the fourth month of gestation, the retina remains avascular as the hyaloid vasculature provides nutrients to the developing retina. Later, the hyaloid artery regresses to the optic fissure. In the fourth month of gestation, the first retinal vessels appear when solid endothelial cords sprout from the optic nerve head to form the primitive central retinal arterial system. The formation of retinal vasculature continues throughout gestation and achieves the adult pattern by the fifth month after birth [23]. Curiously, the central retinal artery divides into four arterioles to supply the retinal quadrants. Multiple subdivisions follow from each retinal vessel in a manner that the retinal vasculature is unique for each individual, much like a fingerprint [24].

### 2.2. Vascular Anatomy of the Posterior Eye Segments in the Adult

One of the main functions of the choroid vasculature is to deliver oxygen and nutrients to the outer retina [23]. The choriocapillary network has the distinctive premises of lying below the BM and RPE (Figure 1). Choriocapillaries facing the BM are fenestrated which allows easy movement of large macromolecules into the extracapillary compartment. Macromolecules, nutrients, and oxygen fluidly infiltrate through the BM and nourish the RPE through their basal cytoplasmic membrane. On the RPE’s apical border, the movement of the extracapillary fluid is blocked by zonula occludens junctions. Accordingly, the RPE can block the passive flow between the inner and outer retina, effectively acting as a component of the blood retinal barrier [23].

The retinal microvessels originate from the central retinal artery and supply the inner segments of the retina (Figure 1). While the choroidal vasculature endothelium is fenestrated and naturally permeable, the retinal vascular ECs have high resistance junctions—zonula adhaerens—and lack fenestrations. The mentioned characteristics of retinal endothelia are preserved over interactions with other cells of the retina including neurons, glial cells, and pericytes, which together with the RPE provide the blood retinal barrier [25].

## 3. Pathological Neovascularization of the Posterior Eye

In ocular vasculopathies, the vessels become leaky, a result of increased stimuli from VEGF and other inflammatory mediators, which cause alterations of junctions in the retinal endothelium [26]. In the process of pathogenic neovascularization, endothelial sprouting is mediated by ECs as major players in the tip and stalk of the new vessels [27]. Tip ECs migrate to guide the new forming vessel, and stalk ECs proliferate and build the sprout. Subsequently, the formation of lumen occurs, and after recruitment and placement of other vascular cell types—pericytes and smooth-muscle cells—the new vessel is formed [28]. In nAMD, accumulated debris, or drusen deposits, activate a molecular signaling flow with a consequence of new capillaries growth from the choroid towards the retina [29]. In PDR, the presence of toxic metabolites causes destabilized conditions in the retinal vasculature with subsequent oxygen limitation in the tissue in which hypoxia triggers neovascularization in the retina [30].

### 3.1. Different Clinical Pathological Conditions: PDR and nAMD

The most challenging ocular angiogenic conditions in the modern world, such as PDR and nAMD, are associated with retinal neovascularization (RNV) and CNV [31]. RNV is recognized as vessel sprouts beginning in the retinal capillaries, then invading into the vitreous and neural layers of the retina. CNV sprouts from the choroidal vessels, which invade the subretinal space. RNV is frequently detected in the course of both PDR and retinopathy of prematurity (ROP), while CNV occurs in AMD patients [32,33].

Macular degeneration is identified as an age-related disease, carrying a high risk of blindness in non-treated cases. According to severity, AMD has been categorized as early, intermediate and late, where late AMD additionally subdivides into dry and wet forms [1]. Dry AMD is recognized as geographic atrophy and non-neovascular forms, and neovascular or wet AMD is identified as neovascularization in the choroid [5].

Generally, diabetic retinopathy (DR) is fairly frequent among adult diabetic persons and in progressive conditions has been reported to damage the retina [34]. DR is described as microvascular malfunctions during diabetes, and is recognized in three forms: diabetic maculopathy, background retinopathy, and PDR [35,36]. PDR is accompanied with the formation of new leaky micro-vessels in the retina, prone to bleeding, which results in vitreous hemorrhage, fibrosis, and ultimately retinal detaching.

## 4. Therapeutic Strategies for Eye Neovascularization

While RNV and CNV initiate in different vascular networks and affect different layers in the retina, clinical treatment of both pathological angiogenesis has been similar [1,37]. More classically, both are addressed by laser photocoagulation, albeit with different clinical approaches using lower energy laser treatments and panretinal targeting to ablate the vasculature in RNV, while higher energy laser is used in CNV. Of relevance, corticosteroids have been used to control inflammation associated with both PDR and AMD, with clinical benefits on reducing vascularization. More recently, the introduction of anti-VEGF immunostrategies in the treatment of ophthalmic pathologies has been largely used to address a myriad of ocular angiogenic pathologies.

### 4.1. Classic Therapeutic Methods: Photocoagulation

A common intervention for the treatment of PDR is photocoagulation, using a laser treatment of panretinal photocoagulation. The laser injection generates laser-scars at the retina with the goal of reducing neovascularization [38]. Despite the clinical effectiveness of photocoagulation in halting angiogenesis, these treatments can cause undesirable side effects, including pain during application for the patient, long-lasting retinal scarring, and the possibility of declined peripheral vision. In contrast, focal photocoagulation is applied to CNV membranes, which slows the progression of neovascularization and visual loss. Nonetheless, laser treatment of nAMD has been associated with higher risk of visual loss after treatment in patients with subfoveal AMD and can result in disciform scaring of the choroid [39].

### 4.2. Ophthalmic Corticosteroids

The use of corticosteroids, among which dexamethasone (Ozurdex^®^), fluocinolone acetonide (Retisert^®^) and triamcinolone acetonide (Triesence^®^), have been approved for the clinical treatment of PDR and nAMD (reviewed in [40,41]). Such corticosteroids modulate inflammation-mediated neovascularization and have shown relative potency in ameliorating RNV and CNV. Nevertheless, the use of corticosteroids in ophthalmology has displayed clinical differences in efficacy, pharmacokinetics, and safety profiles, associated to each specific molecule administered as well as inter-patient variation to treatment. Presently, corticosteroids as adjuvants in anti-VEGF therapies have been emerging as therapeutic options in neovascular ocular diseases [42].

### 4.3. Anti-VEGF Strategies

As a secreted glycoprotein, VEGF shows proangiogenic properties by binding to its receptors (VEGFR), expressed in the surface of ECs, to promote cellular proliferation and migration. Increased VEGF expression has been reported in RNV and CNV, and plays a critical role in either pathogenesis [43]. Different therapeutic methods have been investigated in previous studies, which include anti-VEGF agents that prevent the action of VEGF for angiogenesis during hypoxia (reviewed in [44,45]). The anti-VEGF drugs pegaptanib (Macugen^®^), bevacizumab (Avastin^®^), ranibizumab (Lucentis^®^), and aflibercept (Eylea^®^) are clinically used in ophthalmic neovascular pathologies [31]. It is noteworthy to add that pegaptanib is rarely used nowadays, bevacizumab (an anti-VEGF human recombinant antibody approved in oncology) is used as an off-label treatment in ophthalmology, while ranibizumab and aflibercept, a Fab antibody fragment and a VEGFR-IgG chimeric protein respectively, are approved for clinical use in PDR and nAMD. These strategies have shown very promising outcomes in patients’ vision. However, intravitreal injection of anti-VEGFs raises the risk of post-injection, as well as drug-associated, side effects. In addition, repeated long-term injections are necessary for the treatment of ocular neovascularization, which may lead to increased ocular and systemic complications, together with high economic burden [46]. Notably, in some patients, anti-VEGF treatment is not effective, which highlights differences between patients or even between ocular vascular pathologies. These different clinical responses could both depend on the different anatomical availability and possibly also on the different characteristics of the targeted cells; primarily REC in RNV and CEC in CNV.

### 4.4. Clinical Response Differences between RNV and CNV

Bevacizumab is used as an off-label drug in treatment programs for nAMD [47]. Ranibizumab and bevacizumab have been reported to be effective against CNV, and later, together with pegaptanib, both have been documented to be effective against RNV [48,49,50]. Aflibercept is an inhibitor of VEGF initially developed for the treatment of CNV [51], although it is prescribed for diabetic retinopathy (DR), however its effects are not as beneficial as CNV in patients with DR.

An optimal response to anti-VEGF ocular therapy should include both resolution of excess interstitial fluids, including subretinal fluid and intraretinal fluid, resolution of retinal thickening, and improvement of more than five letters, subject to the maximum effect based on starting visual acuity [52]. However, some reports indicated that the efficiency of anti-VEGF therapeutics reduced after long-term treatments. For instance, intravitreal injection of bevacizumab on the third administration decreased to half of the first administration. This condition—tachyphylaxis—can cause the recurrence of neovascularization after treatment with antibodies against VEGF [53]. Hence, studies concentrated on finding new targets for the treatment of RNV and CNV are afflicted by the mentioned challenges, and novel focus on different ECs from their corresponding origin is paramount.

## 5. The Role of Hypoxia in Pathologic Events of RNV and CNV

High levels of oxygen are consumed by the retina, one of the most metabolically active tissues in the human body [54]. The retinal and choroidal circulations are responsible for sustaining the high oxygen levels required by the retina. In that manner, the choroidal vasculature having high vascular density, nourishes the external layers of the retina, including RPE as well as photoreceptors (Figure 1), while the inner layers of the retina are sustained by the retinal vasculature. Retinal ischemia is a key factor in the pathogenesis of both RNV and CNV. The abnormalities in retinal vasculature, hemorrhage, soft exudates, and thickening of the BM can lead to oxygen deficiency and hypoxia, thus initiating elevation in the expression of angiogenesis factors.

It has been reported that the mean oxygen tension is considerably lower in the lens and vitreous cavity of diabetic patients than non-diabetic individuals [55]. Also, the expression levels of hypoxia-mediated factors were higher in preretinal membranes of diabetic mice and rats in comparison with non-diabetic controls [56,57,58]. On the other hand, higher oxygen intake as a consequence of increased metabolic activity of the retina during inflammation or poor blood circulation of the macula due to vessels’ stenosis, as well as microthrombosis, could be the reason for establishment of a hypoxic milieu in AMD [59,60,61]. Thickening of BM, drusen formation, and reactive oxygen species, which collectively stabilize and raise the levels of HIFs—the key transcription activators of hypoxic-mediated angiogenesis signaling—are of particular importance in hypoxia-mediated AMD progression and CNV [62,63]. Additionally, HIF-1α and HIF-2α have been shown to be upregulated in ECs and macrophages in CNV membranes [64,65]. Regardless of the differences in etiology, hypoxia with subsequent neovascularization is the basic factor involved in the pathology of both PDR and nAMD [11].

### 5.1. The Hypoxia-Inducible Transcription Factors

In mammalian embryonic development, the role of HIFs is fundamental as their genetic deletion led to embryonic lethality in mice, and plays an essential role in the regulation of metabolism in humans [66]. The conserved transcriptional complex, HIF-1, is expressed in many species and all cell types. HIF belongs to the basic helix–loop–helix (bHLH) family of transcription factors, and subfamily of Period–Arnt–Sim (PAS). HIF-1 has been described as a heterodimeric protein composed of α- and β-subunits, where the β-subunit in HIF-1 complex is also known as aryl-hydrocarbon receptor nuclear translocator (ARNT) [13]. Both subunits have three main domains: N-terminal bHLH motif; central PAS domain; and C-terminal transactivation domains. The bHLH motif mediates the binding of HIF to DNA, the central PAS domain enables the heterodimerization of the subunits, and the C-terminal domains are responsible for recruitment of other transcriptional co-regulator proteins and activation of transcription [67].

**Figure 2:**
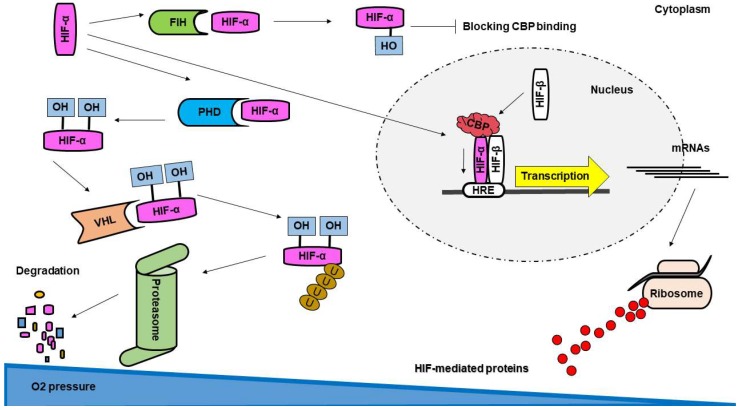
Regulation and function of hypoxia-inducible factors (HIFs). In normal oxygen pressure (normoxia) HIF-α subunits are hydroxylated by prolyl hydroxylase domain (PHD) protein. Hydroxy-HIF-α is recognized by the von Hippel–Lindau E3-ubiquitin ligase complex (VHL), and ubiquitinated HIF-α is subjected to degradation by the proteasome. Simultaneously, HIF-1α is hydroxylated by the factor inhibiting HIF-1 (FIH) protein, resulting in failure to assemble with the transcriptional coactivator CREB-binding protein (CBP). In hypoxia (low oxygen pressure), HIF-α is stabilized, translocates into the nucleus, and dimerizes with HIF-β. Subsequently, HIF heterodimer recognizes the hypoxia-response element (HRE) in the promoter of target genes and assembles with CBP and other coactivators to initiate translation and synthesis of HIF-mediated proteins. OH: hydroxyl; U: ubiquitin.

In normoxia, HIF-α subunits are post-translationally modified by hydroxylation at proline residues (highly conserved among species) by prolyl-hydroxylases (PHD), sanctioning it to recognition by a ubiquitin ligase (von Hippel–Lindau; VHL-E3 complex) with consequent addition of ubiquitin tree and degradation by the proteasome complex (Figure 2) [68]. Additionally, a hydroxylation of an asparagine residue within the C-terminal transactivation domain of HIF-α is introduced by the factor inhibiting HIF-1 (FIH-1), which impairs the recruitment of the coactivator cAMP response element binding protein (CBP) [69] and blocks the hypoxia signaling (Figure 2). Both PHDs and FIH-1 are considered oxygen sensors, as they require molecular oxygen as enzymatic subtract to perform HIF-α hydroxylation and negative regulations [70]. As such, in hypoxia the dioxygenases become inactive and HIF pathway is initiated. HIF-β subunits are constitutively expressed, independent of oxygenation, and during oxygen deprivation the stabilized HIF-α subunits shift to the nucleus to heterodimerize with HIF-β. In the nucleus, HIF transcription factors recognize NCGTG sequence on promoters of target genes, and through recruitment of transcriptional coactivators, initiate the survival of cells in hypoxic conditions (Figure 2) [11], via upregulation of several genes including angiogenesis growth factors such as VEGF, EPO, and anaerobic metabolism (glycolysis and lactate) [12].

### 5.2. HIF-Mediated Genes in RNV and CNV

Decreased oxygen supply in retinal tissues results in activation of HIFs. Evidence shows that HIF-1α, is highly expressed in the retina [71]. In addition to VEGF, EPO and glycolytic enzymes, HIFs upregulate genes encoding factors responsible for regulating matrix degradation, such as urokinase-type plasminogen activator receptor (uPAR), matrix metalloproteinases (MMP), and plasminogen activator Inhibitor-1 (PAI-1) [69], which effectively contribute to ECs proliferation and migration during HIF-mediated angiogenic responses. Other HIF target genes with downstream angiogenesis effectors, particularly on ECs, include among others: angiopoietin-2 (Ang-2) for endothelial sprouting; stromal cell-derived factor-1 (SDF-1), platelet-derived growth factor-B (PDGF-B), insulin-like growth factor-2 (IGF-2), IGF binging proteins (IGFBP) for ECs proliferation and maturation; and C-X-C chemokine receptor 4 (CXCR4) for migration and invasion [11]. The expression patterns of HIF-mediated genes have been shown to differ in CNV and RNV (reviewed in [11]), which correlates to differences in REC and CEC responses and their involvement in PDR and nAMD pathologies.

## 6. ECs in Angiogenesis

Angiogenesis is a process in which new blood vessels are generated from pre-existing ones. This is a physiological process in biological systems and is orchestrated by stimulation of ECs to proliferate and migrate. In pathology, angiogenesis is associated with a myriad of diseases, and in many cases associated with ischemia and hypoxia. In ophthalmic diseases, neovascularization often results in decreased vision, concomitantly with metabolic and cellular dysfunction of ECs. With regards to retinal and choroidal patho-angiogenesis, such as PDR and nAMD, REC and CEC are stimulated into sprouting angiogenesis and result in RNV and CNV. Despite being fundamentally similar, the molecular and signaling pathways differ in RNV and CNV [1], illustrating differences between REC and CEC. In that manner, the isolation and characterization of REC and CEC, together with identification of their differential properties, would be pivotal in understanding the molecular events leading to RNV and CNV.

### 6.1. EC-Based in Vitro Angiogenesis

Animal models of angiogenesis have been employed in research. However, due to complexity, high cost, time consuming procedure, ethical issues linked to animal use, and priority for checking human specific responses, researchers have invested in developing alternative in vitro models [72]. EC culture models are advantageous for the study of hypoxic and angiogenic conditions as they allow control and to specifically manipulate external interfering factors, granting higher reproducibility in angiogenic studies [73]. Beyond reproducibility, in vitro experiments have the advantages of lower costs, shorter times, and specific control of the parameters due to fewer numbers of independent variables [73,74]. Additionally, in vitro cellular models can be employed to assess different combinations of experimental parameters, often not applicable in animal models, due to experimental variation and ethical restrictions [75].

The ECs utilized for in vitro models can be immortalized cell-lines or primary cells [76,77]. Primary cells show limited cell division numbers, usually become non-proliferating or senescent within a few population divisions, and also denote inter-isolate variations. In comparison, immortalized cell-lines generally grow faster, for greater passage numbers, although they may exhibit altered growth features, display tumorigenic potential with chromosomal aberrations, and secretion or expression of many tissue-specific factors can be decreased [72]. Thus, primary cultures of ECs are preferable in neovascularization research since they more closely relate to the physiology, function and metabolic activity of their native counterparts. The first isolated ECs—human umbilical vein endothelial cells (HUVEC) [78]—are nowadays well characterized and widely utilized in in vitro models of angiogenesis. They have been employed in various studies due to availability of umbilical veins, the simple isolation protocol, and the high purity of the isolated cultures [79].

Interestingly, it has been documented that EC phenotypes differ among diverse organs/tissues, and even different segments of the vasculature within the same tissue/organ [7]. Hence, the use of HUVECs may not be truly illustrative for the investigation of the involvement of ECs in pathophysiological mechanisms originating from ocular blinding diseases [15]. In this regard, to clarify the exact mechanisms of pathologic conditions of eye angiogenesis it would be beneficial to study the functions of ECs derived from the tissues where the disorder arises, particularly when the purpose is to translate preclinical findings to clinical practice [80].

### 6.2. Isolation and Characterization of ECs from Retinal or Choroidal Vasculature

Isolation and culture of ECs has been performed from different sources and species, including microvascular or macrovascular endothelium from bovine, murine, and human vessels [81]. In ophthalmic research, retinal and choroidal microvascular endothelia are fundamental parts in the development and progression of RNV and CNV, yet the etiological mechanisms have not been fully understood. Isolation and characterization of angiogenic profiles of both REC and CEC individually would benefit in the molecular studies of PDR and nAMD.

Primary REC and CEC provide an appropriate model of vascular endothelium for investigations of the alterations of genes and proteins expression, as well as their responses to environmental stimuli that mimic the eye neovascularization milieu [82]. Proper isolation of REC versus CEC is very dependent on a careful and precise dissection of both the retina and choroid. Care should be taken as separation of the retina and the choroid is difficult as a consequence of the fragility of the tissues. In addition, microvascular ECs suffering from mechanical damage to the tissue, are sensitive to enzymatic digestions, and the complexity of eliminating other contaminating cells [83]. Pericytes, fibroblasts, and RPE cells are the major groups of cells that contaminate primary ocular EC cultures [84,85]. Of note, ocular ECs may differentiate into fibroblastic cells after several subcultures, and the use of primary eye ECs for in vitro studies should be performed with early culture passages [86].

The techniques used for isolation and purification of REC and CEC have similar procedures, which include several common steps, often starting with manual dissection, subsequent enzymatic digestion with collagenase/dispase enzyme combinations, and use of cell-strainer meshes for debris removal and single-cell suspension. At this step, isolation of EC from the remainder cell-types is generally performed taking advantage of the EC-specific surface marker—cluster of differentiation 31 (CD31) or platelet endothelial cell adhesion molecule-1 (PECAM-1)—using antibody-coated magnetic beads or fluorescence activated cell sorting (FACS) [6,15,87]. Decontamination of EC cultures from other cell types routinely follows protocols of selective-trypsinization and -seeding, or the use of cell-specific coating subtracts and selective cellular inhibitors (e.g., fibroblast inhibitors). Alongside isolation and decontamination techniques, growth media, supplements, and plate surface coating may affect the transcriptome or proteome expression profile of EC cultures. However, there is limited evidence of which technique is preferable, as some methods might affect the expression of genes or proteins intended for investigation.

In vitro cultures of ECs show various morphologies, yet at full confluence the cobblestone morphology is a hallmark characteristic of pure ECs in culture [88]. Uptake of acetylated low density lipoprotein (ac-LDL) by scavenger-cell pathways is a characteristic of ECs and macrophages [89]. Thus, cobblestone morphology observation and ac-LDL uptake assay are standards to characterize ECs without pericyte, smooth muscle, and macrophage contamination. In a comparative study, immunofluorescence staining of ECs markers including PECAM-1, von Willebrand factor (vWF), and isolectin along with checking morphology identified no significant difference between REC and CEC [6]. Moreover, it has been shown that vWF, CD31, CD105, VEGFR1, and VEGFR2 are comparable in REC and CEC but CD34 expression was reported to be higher in REC than CEC [15,90].

### 6.3. Culturing ECs under Hypoxia in Vitro

Routinely, the expansion procedures for ECs are established at room oxygen concentrations, exposing ECs to an average of 20% oxygen (normoxia) [15]. In such culture conditions, partial oxygen pressure is 152 mmHg compared to the partial pressure in arterial blood of 100 mmHg, equivalent to 13.1% oxygen [91]. On the contrary, a hypoxic microenvironment where HIF-1α protein is detectable begins at approximately 5% and reaches its maximum around 1% oxygen concentration [92]. Considering such facts, monitoring oxygen during an experiment with ECs should ensure equal oxygen levels to avoid bias and to guarantee experimental reproducibility [93].

A number of methods have been employed for hypoxia set up in different studies mainly including volume restriction, biochemical hypoxia, and atmospheric hypoxia. McLeod and colleagues used volume restriction by culturing ECs on microcarrier beads, in order to restrict oxygen together with medium volume, by allowing the ECs to sediment in a test tube [94,95]. Another strategy for generation of the hypoxic niche is the addition of chemical compounds which induce intracellular hypoxia-mimicking conditions. In such strategies, the aqueous solution resides oxygenated and the chemical compound treatments provide oxygen deprivation conditions or modulate the hypoxia-associated signaling, with resulting stabilization of HIF-1α protein.

HIF dioxygenases—PHDs and FIH-1—use divalent iron (Fe^2+^), oxoglutarate, and oxygen as substrates to hydroxylate HIF-α subunits. Chemical hypoxia-mimicking agents focus on rendering the dioxygenases inactive by competing with its substrates [96,97,98,99,100,101]. Nickel chloride, as well as the extensively used cobalt chloride (CoCl_2_), directly compete with Fe^2+^. Quelating agents, such as deferoxamine and 2,2′-dypiridyl, function as stabilizers of HIF-α by chelating the iron core [98,102]. Additionally, chemical compounds, with the example of dimethyl-oxalylglycine (DMOG), stabilize the HIF-1α by inhibition of the dioxygenases by substituting oxoglutarate [103]. However, these biochemical methods confine the hypoxia study to downstream of HIF since they unsuccessfully reproduce the signaling of mitochondrial ROS mediated by hypoxia [104,105]. Ideally, in vitro hypoxia should be enabled by introducing other gases (mainly nitrogen) which replace oxygen molecules in sealed-atmospheric chambers [96]. Both gas-controlled incubators and chambers have limitations regarding methods that require transition of the cell cultures into ambient atmosphere (21% oxygen), resulting in an interruption of the hypoxia and eventual hypoxia-mediated pathways.

## 7. Gene and Protein Expression in REC versus CEC

Understanding the phenotypical differences between REC and CEC is essential for treatment of specific eye vasculopathies, which guarantees effective targeting of pathogenic neovascularization without side effects to other vasculatures. Differential analysis of mRNA and protein expressions of REC and CEC should be considered without any external factors at baseline, or following external stimuli, such as hypoxia, which would resemble more closely molecular events in pathologic ocular angiogenesis.

### 7.1. Baseline Differences in the Molecular Profiles of REC and CEC

To date, studies aimed at characterizing the molecular phenotype of human REC and CEC are very few, in part due to difficulties in isolation, culturing, and donor limitations. A major restriction in comparative analysis is possible inter-donor variations. One solution to overcome this problem is donor-matched profiling of REC against CEC, as previously proposed [6].

Molecular phenotype could be defined by gene transcription and protein expression of the ECs. It is not surprising that REC and CEC have many common similarities attributable to their endothelium origin. With focus on donor-matched differences in molecular phenotypes, rather than similarities, Mammadzada et al. analyzed CEC compared to REC using genes relevant in angiogenesis and biology of ECs [6]. The results revealed a multitude of genes differentially expressed in REC compared to CEC, including up and downregulated genes. The upregulated transcripts included mRNAs for the proteins C–C motif chemokine ligand 2 (CCL2), C–X–C chemokine ligand (CXCL16), MMP9, prostaglandin synthase 1 (PTGS1), vascular cell adhesion protein1 (VCAM1), and the highest fold-increase for interleukin 7 (IL7). While downregulated genes in CEC normalized to REC were represented by angiopoietin-like 4 (ANGPTL-4), collagen type IV alpha 3 (COL4A3), endothelin 1 (EDN1), endothelin receptor type A (EDNRA), coagulation factor II receptor (F2R), coagulation factor III (F3), pigment epithelium derived factors (PEDF), natriuretic peptide B (NPPB), TEK tyrosine kinase (TEK), VEGF-C, connective tissue growth factor (CTGF), fibroblast growth factor 1 (FGF-1), transforming growth factor alpha (TGFα), integrin β3, and the highest downregulated mRNA was reported to be placental growth factor (PlGF) [6]. In general, genes involved in cell proliferation and vessel maturation had less transcripts in CEC than in REC, whereas cytokines and chemotactic cytokines, ECM degradation, inflammatory prostaglandins, and cell adhesion and migration genes showed higher mRNA levels in CEC as compared to REC [6]. Similar studies have reported less expression of VEGF in CEC versus REC [16], where VEGF-165 or -121 isoforms were more influential on REC proliferation than in CEC [90]. The significant higher transcription of IL7 in CEC than REC illustrates possible mechanisms by which CEC could direct angiogenesis via cytokines [6], with putative enrolment of IL7 receptor (IL7R) expressed in human microvascular ECs [106]. Conversely, REC appear to drive neovascularization through PlGF-centered actions and its receptor VEGFR1 [6].

A microarray profiling has been conducted on donor-matched samples of human primary REC and CEC [107], and the results revealing a distinct profile for each EC-type. Interestingly, the differences between the two cell-types were reported to be more considerable than inter-donor variations, which denote the heterogeneity of the two vascular beds. Besides, a gene ontology study showed that approximately 9% of differentially expressed genes in REC and CEC belong to genes involved in cell proliferation, implying angiogenesis in the particular case of ECs [18]. Moreover, REC exhibited higher levels of immunologic response or inflammation related genes [107]. Confounding, in bovine REC transcripts encoding for CCL5, granulocyte-macrophage colony stimulating factor (GM-CSF), and macrophage colony stimulating factor (M-CSF) were downregulated compared to CEC [3], a finding that may innately accommodate some inter-specie differences.

At the protein level, the expression status between REC and CEC populations strongly supports the hypothesis of differential regulation of angiogenesis in these two EC-types. An in vitro proteome study in REC detected several proteins with a role associated with ocular angiogenesis with significant fold difference to CEC, particularly two secreted proteins—netrin-4 (NET4) and thrombospondin domain-containing protein 4 (THSD4)—and one cytoskeleton protein, Testin [18]. Netrin ligands and receptors are recognized for their potential in both formation of neuron network and vasculogenesis during development [108]. NET4, as a member of this family, was found in retina and its expression is highly enhanced in VEGF-stimulated ECs, both in vitro and in the laser-induced CNV model [109,110,111,112]. THSD4 is involved in ECs interaction with ECM, mandatory during blood vessel sprouting [113,114]. Curiously, Testin has been detected in focal adhesions and can prevent angiogenesis, suggesting that neoangiogenesis in REC might be more tightly regulated than in CEC [18,115].

By comparison, proteins involved in angiogenesis including actin-binding protein anillin, nesprin-3, and neural precursor cell expressing developmentally downregulated protein 4 (NEDD4) were reported to be conceivably more relevant in CEC than REC [18]. The intracellular scaffold-protein anillin contributes to cytokinesis during cell division [116] and may be involved in neovascularization processes from the choroid. Nesprin-3 has been reported to be involved in organelle positioning, divisions, polarity, and migration of ECs [117], with particular relevance in choroid neovascularization [18]. Functionally, NEDD4 contributes in p38 mitogen-activated protein kinase signaling, involved in elevated permeability of vasculature [118], suggesting that NEDD4 upregulation in CEC may promote leakage observed during the neovascularization in nAMD [18].

Together, the gene arrays and proteomic analysis results determine that the baseline expression of angiogenic factors differs broadly between REC and CEC (Table 1), highlighting variation within ocular ECs.

### 7.2. Differences in Molecular Profile of REC and CEC in Response to Hypoxia

The hypoxia niche and subsequent activation of HIF signaling are responsible for regulating multiple aspects of angiogenesis, such as ECs activation in both the retina and choroid. Study of hypoxia as an angiogenesis-stimulating factor provides new insights for anti-angiogenesis therapy approaches in PDR and nAMD.

**Figure 3:**
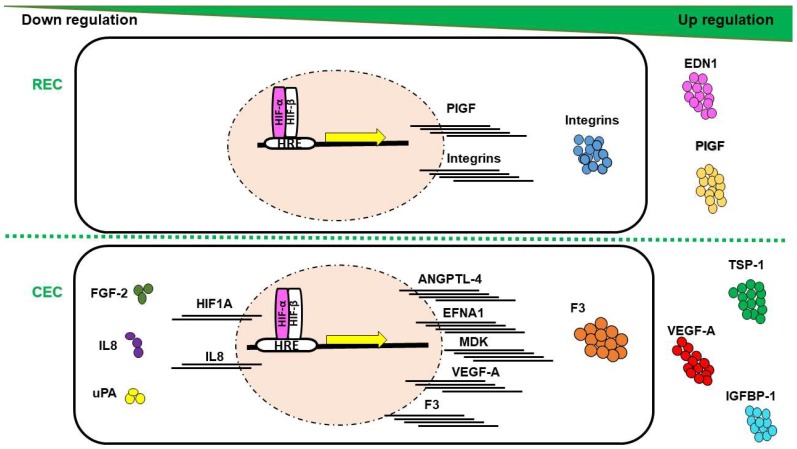
Schematic depiction of angiogenesis-related mRNA, and intracellular and soluble protein expression profile in response to hypoxia (yellow arrow) compared between retinal endothelial cell (REC) and choroidal endothelial cell (CEC). Lines: mRNAs; circles: proteins.

Evaluation of HIF-α subunits’ expression in human REC and CEC exposed to hypoxia showed maximum upregulation of both HIF-1α and HIF-2α after 6 h of in vitro hypoxia, equally in both EC-types from human [6] or bovine origin [3]. The main downstream targets of HIF with immense effect on pathologic neovascularization are the VEGFs. A number of proteins that contribute in the complex process of angiogenesis compose the VEGF family, including VEGF-A, B, C, D, E, F, and their analogue PlGF. The upregulation of VEGF-A and PIGF plays a central role in the pathogenesis of PDR and nAMD [119,120]. Previous work has shown that the expression of VEGF-A mRNA and VEGF-A protein secretion is significantly higher in CEC exposed to hypoxia, while REC upregulates the transcription, translation and secretion of PlGF (Figure 3) [6]. Similar studies, showed increased expression of VEGF-A and VEGF-C in HUVECs exposed to hypoxia [121], while the proangiogenic protein PlGF expression was more canonically upregulated in lymphatic ECs upon hypoxia [122].

In response to hypoxia, mRNAs for the proteins ANGPTL-4, ephrin A1 (EFNA1), and midkine (MDK) have been shown to be upregulated in CEC compared to REC (Figure 3) [6]. ANGPTL-4 and MDK can interact with the ECM of ECs with pleiotropic effects in hypoxia-mediated angiogenesis, such as promotion of cell proliferation and migration [123,124,125,126]. EFNA1 has been reported to contribute to vascular remodeling through specific mediation by HIF-2α [127]. This could be related to the slight decrease in *Hif1a* transcripts as previously reported [6], suggesting an activation of HIF-2α by CEC in adaptation to hypoxia.

At the protein level, F3, thrombospondin-1 (TSP-1) and IGFBP-1 were upregulated in hypoxic CEC in comparison to REC (Figure 3) [6]. These proteins orchestrate responses in EC proliferation, vessels stabilization, smooth muscle cells recruitment, and have been detected with increased expression levels in nAMD membranes [128,129,130,131,132,133]. Noteworthy, F3 and IGFBP-1 have been reported to be overexpressed in hypoxic milieu yet regulated independently of HIFs [133,134]. Collectively, hypoxia-mediated upregulation of CEC targets indicates a probable involvement in CNV rather than in RNV. Conversely, EDN1 has been differentially detected in REC compared to CEC under hypoxic conditions (Figure 3) [6], triggering proliferation, migration, and tubulogenesis in ECs, either single-handedly or in combination with VEGF [135]. Several studies have confirmed EDN1 upregulation by hypoxia in REC and to take a part in retinal angiogenesis associated with PDR [136,137], through a HIF-1 binding site on EDN1 promoter [138]. Moreover, EDN1 has been shown to stabilize HIF-α subunits by PHD2 inhibition [139], which could be associated with longer length of expression of HIF-1α and -2α in REC as compared to CEC [6]. Members of the integrin family (integrin αv, β3, and β5) expression at mRNA and protein levels were upregulated in bovine REC exposed to hypoxia (Figure 3), via an autocrine/paracrine activity concomitantly with VEGF yet independently of HIFs activity [140].

Interestingly, FGF-2, IL8 and urokinase-type plasminogen activator (uPA) have been shown to be downregulated in CEC (Figure 3), while unmodified in REC, in response to hypoxia [6]. FGF-2 facilitates angiogenesis, yet can suppress endothelial function particularly in models of retinal ischemia [141], findings paralleled by IL8 [142]. It has been shown that hypoxic conditions could modulate uPA mRNA and protein levels in vitro [143]. Consistent with such findings, a study conducted on ECs from bovine pulmonary microvasculature demonstrated a decrease in uPA expression in hypoxia [144]. Thus, downregulation of angiogenic factors in CEC may emphasize their negative regulatory role in CNV.

Despite similar levels of expression of HIF-α subunits in REC and CEC, the hypoxia-mediated signaling differs in choroidal and retinal tissues. Multiple angiogenic factors and cytokines are differentially expressed in RNV and CNV (Table 2), and may illustrate the considerable differences in PDR and nAMD progression.

### 7.3. Differences in Molecular Profile of REC and CEC in Response to External Stimuli Other Than Hypoxia

Understanding the responses of REC and CEC to various in vitro treatments can define their effects on the function of ECs and significantly contribute to clarifying the pathophysiological modifications detected in different diseases in the eye.

ECs can vary in their reactions to external stimuli, as glucose promotes the secretion of plasminogen activator by REC, whereas HUVECs do not show related responses [84]. REC and CEC treated with high glucose concentrations in culture displayed increased permeability, with a greater impact on REC [145]. At high glucose concentrations, the expression of occludin, claudin-5, and junctional adhesion molecule A (JAM-A) were downregulated in REC, yet vascular endothelial (VE)-cadherin and JAM-C showed similar expression between the two ECs. Thus, the control of permeability by junctional molecules is not identical between REC and CEC.

Browning et al. investigated the proliferative and sprout formation effect of VEGF-165, FGF-2, IGF-1, PDGF-AA, PDGF-BB, and IL1β on the human macular inner CECs. Both VEGF-165 and FGF-2 considerably increased CEC proliferation and sprout formation [14]. Thus, targeting growth factors other than VEGF, such as FGF-2, may be of particular interest in the therapeutic plans for CNV rather than RNV.

Prevention of VEGF effects in terms of angiogenesis has proven to be an outstanding strategy in anti-angiogenesis therapy, particularly in tumor and ocular pathologies. In this way, the effect of VEGF inhibitors (pegaptanib, bevacizumab, ranibizumab) on primary cultures of human REC and CEC was evaluated by Stewart et al. [90]. Significant differences between retinal and choroidal microvascular cells were observed, which highlights the need for specific treatment interventions in RNV or CNV. Stimulation with two VEGF isoforms 121 and 165 were potent on REC and CEC proliferation, albeit REC presented higher proliferation when compared to CEC. Ranibizumab and bevacizumab decreased proliferation of CEC promoted by both VEGF isoforms, either in combination or separately, while ranibizumab was moderately more active, in REC [90]. Such findings deepen the clinical relevance of addressing RNV and CNV separately (Table 3).

## 8. Future Therapeutic Strategies for Ocular Neovascularization

Ample evidence has accumulated to illustrate phenotypical differences and the differential expression patterns of multiple angiogenic factors in RNV and CNV. Nevertheless, the current clinical approach for the treatment of both PDR and nAMD is paralleled with photocoagulation and anti-VEGF agents. Future clinical treatments could address upregulated factors specifically for either ocular pathology, or the use of broader therapeutic agents that modulate multiple pathways or milieu-driven responses.

### 8.1. Anti-HIF Gene Therapy

Undoubtedly, HIFs are pivotal in hypoxia-mediated responses of ECs, including REC and CEC [6], and evidence of the role of HIFs in posterior eye segment diseases is increasing [4,11,13]. Animal models of genetic modulation of the HIF pathway within retinal cells have shown vascularization alterations [146,147]. In mouse models of retinal chronic hypoxia, gene therapy silencing HIF-1α by interfering RNA recovered the retinal phenotype [148]. In a mouse model of nAMD, negative regulation of HIFs has been achieved by gene transfer of PHD2 with significantly reduction of CNV [149].

Nevertheless, genetic ablation of HIFs and HIF-mediated responses should be taken with care, since it would dramatically impair the ability of ocular tissues to tolerate and adapt to hypoxia [6]. As such, anti-HIF gene therapy would benefit from cell-type specific expression (expression driven by specific promotors), as well as regulatory elements to promote hypoxia-mediated expression of the therapeutic transgenes [150,151].

### 8.2. Combined Therapies and Novel Targets

The intraocular administration of anti-VEGF therapeutics has been the golden standard for management of neovascularization in the retina and choroid. However, some patients fail to respond to anti-VEGF drugs, suggesting that other mediators might be involve in ocular angiogenesis [152]. Therefore, studies are being directed at finding novel targets for ocular neovascularization therapy, with focus on members of the VEGF sub-family, PlGF family, FGF family, EGF family, TGF-β, ANGPTL family, galectin family, integrin super family, PEDF, some cytokines, and MMPs (reviewed in [152]).

Two probable key therapeutic aspects in either RNV or CNV are differential expressions of specific inflammatory and angiogenesis factors. Li et al., used a strategy based on a bispecific molecule with the potential to suppress both inflammation and neovascularization in CNV, using gene therapy [153]. Despite targeting CNV, the same approach could readily be applied in RNV taking advantage of tissue-specific promoters.

In addition, novel molecules such as UPARANT (designated cenupatide in the International Non-proprietary Names nomenclature) have displayed immense potential to halt angiogenesis in animal models of oxygen-induced retinopathy and laser-induced neovascularization, hence RNV and CNV respectively [154,155]. The intracellular signaling cascade by uPAR is mediated through formyl peptide receptors (FPR), as co-receptor binding in ECs [156,157]. UPARANT is an inhibitor of uPAR/FPR co-receptor binding, canonical in uPA-activated ECs. Moreover, FRPs crosstalk with VEGFRs and integrin signaling pathways [158], which leads to activation of HIFs, STATs, NF-κB, and CREB transcription factors, involved in the upregulation of multiple of angiogenesis and inflammatory factors [159,160,161]. Albeit not yet approved for clinical use, UPARANT could simultaneously interject multiple proangiogenic factors in ECs, with minimal side-effects on other cells and tissues, which could significantly improve anti-neovascularization treatments of future use in clinic [155].

Similar to UPARANT in its preclinical stages, novel selective corticosteroid receptor agonists, such as the example of Mapracorat, have reveled benefits in ocular animal models, by avoiding side effects of classic corticosteroid treatments [162,163], and augments the use of corticosteroids in ocular neovascular diseases. In addition, novel pathways associated with mechanisms of angiogenesis with relevance for ocular endothelial cells have been highlighted. Of particular interest, the phospholipases pathway has been demonstrated as a potential target in managing PDR, both in vitro and in vivo [164,165], with some modest improvement in a clinical trial, particularly as an adjuvant to anti-VEGF therapies [166]. Together, the preclinical benefits of specific adjuvants to anti-VEGF therapies in PDR and nAMD, further contribute to the differential responses of REC and CEC to angiogenic stimuli.

## 9. Conclusions

This review compared and discussed the differential transcriptome and proteome levels of REC and CEC, two distinct microvascular ECs originating from posterior eye tissues, the retina and the choroid, respectively. RNV and CNV are distinctively driven by angiogenic stimuli, particularly hypoxia, culminating in PDR or nAMD. The presence of critical differences in the molecular profile of REC and CEC in response to hypoxia involves multiple networks, including the VEGF family, chemotaxis genes, cell proliferation and migration, and other inflammation factors. The differences of expression of proangiogenic factors and cytokines seem to be exclusively dependent on the vascular beds from which each EC-type originates. Ultimately, understanding the differential expression patterns of REC and CEC could have perceptive implications for future targets for treatments in the clinic.

## Figures and Tables

**Table 1:** Summary of differential molecular profile in retinal endothelial cell (REC) versus choroidal endothelial cell (CEC).

Endothelial Profile	Symbol	Factor Name	Molecule	Reference
Upregulated in REC vs. CEC	ANGPTL4	Angiopoietin-like 4	mRNA	[6]
	COL4A3	Collagen type IV alpha 3	mRNA	[6]
	CTGF	Connective tissue growth factor	mRNA	[6]
	EDN1	Endothelin 1	mRNA	[6]
	EDNRA	Endothelin receptor type A	mRNA	[6]
	F2R	Coagulation factor II receptor	mRNA	[6]
	F3	Coagulation factor III	mRNA	[6]
	FGF-1	Fibroblast growth factor 1	mRNA	[6]
	ITGB3	Integrin beta 3	mRNA	[6]
	NET4	Netrin-4	Prot	[18]
	NPPB	Natriuretic peptide B	mRNA	[6]
	PEDF	Pigment epithelium derived factor	mRNA	[6]
	PlGF	Placental growth factor	mRNA	[6]
	TEK	TEK tyrosine kinase	mRNA	[6]
	Testin	Testin	Prot	[18]
	TGFα	Transforming growth factors alpha	mRNA	[6]
	THSD4	Thrombospondin domain-containing protein 4	Prot	[18]
	VEGF-C	Vascular endothelial growth factor C	mRNA	[6]
Upregulated in CEC vs. REC	CCL2	C-C motif chemokine ligand 2	mRNA	[6]
	CCL5	C-C motif chemokine ligand 5	mRNA	[3]
	CXCL16	Chemokine (C-X-C motif) ligand 16	mRNA	[6]
	GM-CSF	Granulocyte-macrophage colony stimulating factor	mRNA	[3]
	IL7	Interleukin 7	mRNA	[6]
	M-CSF	macrophage colony stimulating factor	mRNA	[3]
	MMP9	Matrix metalloproteinase 9	mRNA	[6]
	NEDD4	Neural precursor cell expressed developmentally downregulated protein 4	Prot	[18]
	Nesprin-3	Nesprin-3	Prot	[18]
	PTGS1	Prostaglandin synthase 1	mRNA	[6]

Expression determined by transcript (mRNA) or protein (Prot).

**Table 2:** Summary of angiogenesis-related factor regulation in REC and CEC in response to hypoxia.

Biological Process	Symbol	Factor Name	Molecule	CEC	REC	Ref
Cell proliferation and vessel maturation	ANGPTL4	Angiopoietin-like 4	mRNA	↑	–	[6]
	EDN1	Endothelin 1	Prot	–	↑	[6]
	EFNA1	Ephrin-A1	mRNA	↑	–	[6]
	F3	Coagulation factor III	mRNA/Prot	↑	–	[6]
	FGF-2	Fibroblast growth factor 2	Prot	↓	–	[6]
	HIF-1α	Hypoxia-inducible factor 1 alpha	mRNA	↓	–	[6]
	IGFBP-1	Insulin-like growth factor-binding protein 1	Prot	↑	–	[6]
	IGFBP-3	Insulin-like growth factor-binding protein 3	Prot	↑	↑	[6]
	MDK	Midkine	mRNA	↑	↑	[6]
	PlGF	Placental growth factor	Prot	–	↑	[6]
	TSP-1	Thrombospondin 1	Prot	↑	–	[6]
	VEGF-A	Vascular endothelial growth factor A	mRNA/Prot	↑	–	[6]
Chemotaxis and cell migration	CXCL16	Chemokine (C-X-C motif) ligand 16	Prot	↓	↓	[6]
	IL8	Interleukin 8	mRNA/Prot	↓	–	[6]
	ITGAN	Integrin alpha niu	mRNA/Prot	–	↑	[140]
	ITGB3	Integrin beta 3	mRNA/Prot	–	↑	[140]
	ITGB5	Integrin beta 5	mRNA/Prot	–	↑	[140]
	PTX3	Pentraxin 3	Prot	↓	↓	[6]
	uPA	Plasminogen activator, urokinase	Prot	↓	–	[6]

Regulation depicted as ↑/↓/– indicating respectively upregulation/downregulation or no change in response to hypoxia as determined by transcript (mRNA) or protein (Prot).

**Table 3:** Summary of angiogenesis-related factors regulated in REC and CEC in response to non-hypoxia stimuli.

Biological Stimulus	Symbol	Factor Name	Molecule	CEC	REC	Reference
High glucose	Claudin-5	Claudin-5	Prot	–	↓	[145]
	JAM-A	Junctional adhesion molecule A	Prot	–	↓	[145]
	Occludin	Occludin	Prot	–	↓	[145]
EC proliferative factors	FGF-2	Fibroblast growth factor 2	Prot	↑	–	[14]
	VEGF-A	Vascular endothelial growth factor A	Prot	↑	–	[14]

Regulation depicted as ↑/↓/– indicating respectively upstimulation/downstimulation or no change in response to stimulus associated with protein function (Prot).
